# Green Tea Consumption and Depressive Symptoms among Japanese Workers: The Furukawa Nutrition and Health Study

**DOI:** 10.3390/nu14010167

**Published:** 2021-12-30

**Authors:** Akiko Nanri, Masafumi Eguchi, Takeshi Kochi, Isamu Kabe, Tetsuya Mizoue

**Affiliations:** 1 Department of Food and Health Sciences, International College of Arts and Sciences, Fukuoka Women’s University, Fukuoka 813-8529, Japan; 2 Department of Epidemiology and Prevention, Center for Clinical Sciences, National Center for Global Health and Medicine, Tokyo 162-8655, Japan; mizoue@hosp.ncgm.go.jp; 3 Department of Health Administration, Furukawa Electric Corporation, Tokyo 100-8322, Japan; masafumi.eguchi@furukawaelectric.com (M.E.); takeshi.kochi@furukawaelectric.com (T.K.); 4 Kubota Corporation, Tsukubamirai 300-2402, Japan; isamu.kabe@kubota.com

**Keywords:** depressive symptoms, green tea, Japanese, prospective study

## Abstract

Although several cross-sectional studies have described an inverse association between green tea consumption and depressive symptoms, only one study has prospectively investigated this association. We investigated the cross-sectional and prospective associations between green tea consumption and depressive symptoms in a working population in Japan. Participants were 1987 workers who participated in the baseline survey for a cross-sectional association, and 916 participants who did not have depressive symptoms at baseline who responded to both the baseline and follow-up surveys for a prospective association. Green tea consumption was evaluated with a validated self-administered diet history questionnaire. Depression symptoms were evaluated with the Center for Epidemiologic Studies Depression (CES-D) scale. Multiple logistic regression was conducted to estimate the odds ratio of depressive symptoms based on green tea consumption. In the cross-sectional analysis, green tea consumption was not associated with the prevalence of depression symptoms. Moreover, consumption at baseline was not associated with depression symptoms after 3 years; the multivariable-adjusted odds ratio of depressive symptoms for ≥2 cups/day of green tea was 1.12 (95% confidence interval 0.65–1.91) compared with <4 cups/week after adjustment for covariates including dietary factors (trend *p* = 0.67). Our results suggest that there is no association of consumption of green tea with symptoms of depression in Japanese.

## 1. Introduction

Depression is a common mental disorder worldwide which affects more than 264 million people [1]. The effects of depression may be long-lasting or recurrent, and dramatically affect the ability to function and lead a rewarding life [1]. Furthermore, depression can also lead to suicide. Identifying the determinants of depression for prevention is therefore important. Green tea, which is a widely consumed beverage in Asia, is suggested to exert protective effects against depression. Animal studies have demonstrated that green tea polyphenols have antidepression-like effects [2]. Green tea has potent antioxidant activities in vivo [3] and may thus confer protection against depression by decreasing oxidative stress [4]. Moreover, green tea catechins have been shown to exert anti-inflammatory actions [5] and to lower depression [6]. However, epidemiological evidence for an association between green tea consumption and depressive symptoms [7,8,9,10,11,12,13], psychological distress [14], or mental health [15] is inconsistent, and only one prospective study has been reported [11]. Namely, the Singapore Longitudinal Ageing Study [11] suggested an association between green tea consumption and a decreased risk of depression after a mean of 4 years. In that study, however, only 5% of participant drank green tea on a daily basis. A prospective association between consumption and depression symptoms among populations with high green tea consumption, such as Japanese, therefore remains elusive. In this study, we examined the cross-sectional and prospective association of green tea consumption with depressive symptoms in a working population of Japanese.

## 2. Materials and Methods

### 2.1. Study Procedure and Participants

The Furukawa Nutrition and Health Study is a nutritional epidemiology survey that was performed as part of the Japan Epidemiology Collaboration of Occupation Health Study in workers at a manufacturing company and affiliates in the prefectures of Chiba and Kanagawa, Japan. The survey was held at baseline (April 2012, May 2013) as well as at a 3-year follow-up session (April 2015, May 2016). The study procedure is described elsewhere in detail [16]. The study protocol was approved by the Ethics Committee of the National Center for Global Health and Medicine, Japan (ethical approval number NCGM-G-001140-15), and written informed consent from all participants was obtained before to the survey.

Of 2162 workers who participated in the baseline survey, 11 who did not complete the questionnaires were excluded. Among the 2151 participants, 100 who reported a history of cancer, chronic hepatitis, cardiovascular disease, chronic kidney disease (including nephritis), pancreatitis, or mental disease were further excluded. We additionally excluded 64 participants with missing data on CES-D and covariates used in the analysis, leaving 1987 (1777 men and 210 women) for cross-sectional analysis. For the longitudinal analysis, of 1987 participants, we consecutively excluded 729 who did not participate in the follow-up survey, 339 with depressive symptoms (CES-D ≥16) at baseline, and 3 who had missing data on outcome, eventually leaving 916 participants (817 men and 99 women) for the longitudinal analysis.

### 2.2. Depressive Symptoms

Depressive symptoms were evaluated with a Japanese version [17] of the Center for Epidemiologic Studies Depression (CES-D) scale [18], which was included in the lifestyle questionnaire. Criterion validity of this CES-D scale is well established in Western [18] and Japanese [17] participants. In a validation study for the Japanese version, a CES-D score ≥16 indicated a sensitivity of 88.2% and specificity of 84.8% [17]. Symptoms of depression, the main outcome, were considered to be present when CES-D score was ≥16. In addition, as a secondary outcome, a CES-D score ≥23 was used to show a severe depressive state [19].

### 2.3. Dietary Assessment

Dietary habits in the previous month were evaluated using a validated brief self-administered diet history questionnaire (BDHQ) [20]. Validation of the BDHQ with 16-day weighed dietary records as the objective standard reported a Spearman correlation coefficient for the energy-adjusted intake of green tea (using the density method) in 92 men and women each aged 31–76 years of 0.68 and 0.64, respectively [20].

Regarding green tea, eight response options were available for consumption frequency (none, <1 cup/week, 1 cup/week, 2–3 cups/week, 4–6 cups/week, 1 cup/day, 2–3 cups/day, and ≥4 cups/day). We further divided the participants into three categories by green tea consumption so that the number of participants was as close as possible: <4 cups/week, 4 cups/week to 1 cup/day, and ≥2 cups/day.

### 2.4. Other Variables

The questionnaire elicited information on marital status, living status, night and rotating shift work, job grade, job strain, overtime work, smoking, alcohol consumption, physical activity during work and housework or while commuting to work, sleep duration, leisure-time physical activity, and history of diabetes. Physical activities during work and housework or when commuting and in leisure-time were expressed as the sum of the metabolic equivalent (MET) multiplied by the duration of time (in hours) across each level of physical activity. Psychological work environment was evaluated using the Job Content Questionnaire [21], and job strain score was examined by the standard procedure. Body mass index was analysed using the measured body height and body weight.

### 2.5. Statistical Analysis

Baseline characteristics are expressed as means (standard deviation) for continuous variables and percentages for categorical variables. Trend association between confounding factors and green tea consumption were evaluated by linear regression analysis for continuous variables, treating the ordinal numbers in each category of green tea consumption as a continuous variable, or by the Mantel-Haenszel chi-square test for categorical variables.

To examine the cross-sectional association between green tea consumption and depressive symptoms, multiple logistic regression analysis was conducted to estimate odds ratios of depressive symptoms for the category of green tea consumption, with the lowest category as reference. The first model was adjusted for age (year, continuous), sex, and site (baseline survey in April 2012 or May 2013), and the second model was further adjusted for body mass index (kg/m^2^, continuous), marital status (married or other), living status (alone or not alone), overtime work (<10, 10-<30, or ≥30 h/month), job grade (high, middle, or low), night or rotating shift work (yes or no), job strain (quartile), smoking (never-smoker, ex-smoker, current smoker consuming <20 cigarettes/day, or current smoker consuming ≥20 cigarettes/day), alcohol drinking (nondrinker, occasional drinker, drinker consuming <23 g, 23-<46 g, or ≥46 g of ethanol/day), leisure-time physical activity (METs-hour/week, quartile), sleep duration (<6, 6-<7, or ≥7 h/day), physical activity at work and housework or on commuting to work (METs-hour/day, quartiles), under treatment for or history of diabetes (yes or no), and total energy intake (kcal/day, continuous). In the final model, energy-adjusted intakes (using the density method) of folate (μg/day, continuous), vitamin B6 (mg/day, continuous), vitamin B12 (μg/day, continuous), n-3 polyunsaturated fatty acids (%energy, continuous), and zinc (mg/day, continuous), magnesium (mg/day, continuous), and consumption of soft drinks (<1 cup/week, 1–3 cups/week, or ≥4 cups/week) and coffee (<1 cup/day, 1 cup/day, or ≥2 cups/day) were added to the second model. These nutritional factors are reportedly associated with depressive symptoms [13,22,23,24,25].

For analysis of prospective associations, multiple logistic regression analysis was performed to calculated odds ratio of depressive symptoms evaluated in the follow-up survey for the category of green tea consumption at the baseline survey, using the lowest category as reference. The adjusted models were the same as in the cross-sectional analysis, and CES-D score at baseline was added to the model. Trends in the cross-sectional and prospective associations were evaluated by treating the ordinal numbers for each category of green tea consumption as a continuous variable. For sensitivity analysis, we used the same categories of green tea consumption as the previous studies in Japanese of Niu et al. [12] and Pham et al. [13] (≤1 cup/day, 2–3 cups/day, and ≥4 cups/day) and repeated the analyses. Two-side P values <0.05 were considered as statistically significant. All analyses were conducted using Statistical Analysis System (SAS) software v.9.3 (SAS Institute, Cary, NC, USA).

## 3. Results

Of 1987 participants for the cross-sectional analysis, the percentage of participants who consumed none, <1 cup/week, 1 cup/week, 2–3 cups/week, 4–6 cups/week, 1 cup/day, 2–3 cups/day, and ≥4 cups/day of green tea were 7.8%, 9.9%, 8.4%, 14.4%, 12.9%, 17.4%, 22.1%, and 7.0%. The baseline characteristics of study participants by green tea consumption are shown in Table 1. Participants with greater consumption of green tea were more likely to be older and women and less likely to be shift workers, current smokers, alcohol drinkers, and soft drink and coffee drinkers compared to those with lower consumption. They also had higher intake of total energy, vitamin B6, folate, vitamin B12, n-3 PUFA, magnesium, and zinc.

The odds ratios of depressive symptoms by category of green tea consumption are shown in Table 2. Of 1987 participants included in the cross-sectional analysis, 548 (27.6%) were shown to have depressive symptoms. For the cross-sectional analysis, green tea consumption was not associated with depressive symptoms: after adjustment for covariates including dietary factors, the multivariable-adjusted odds ratio (95% CI) of depressive symptoms for ≥2 cups/day of green tea was 1.29 (0.96–1.73) compared with <4 cups/week (model 3: trend *p* = 0.087).

Of 916 participants without symptoms of depression at baseline, 155 (16.9%) were newly identified to have symptoms in the follow-up survey. As seen in Table 2, no significant association was observed between green tea consumption at baseline and the occurrence of depressive symptoms. The multivariable-adjusted odds ratios (95% CI) of depressive symptoms were 1.00 (reference), 1.09 (0.68–1.76), and 1.12 (0.65–1.91) for <4 cups/week, 4 cups/week to 1 cup/day, and ≥2 cups/day of green tea, respectively (model 3: trend *p* = 0.67). Green tea consumption was also not associated with severe depressive symptoms (CES-D score ≥23).

In a sensitivity analysis of using the same category as the Japanese previous studies, we again observed no association between green tea consumption and depressive symptoms; after adjustment for all covariates, the multivariable-adjusted odds ratios (95% CI) of depressive symptoms at 3 years were 1.00 (reference), 1.09 (0.66–1.79), and 0.99 (0.43–2.31) for ≤1 cup/day, 2–3 cups/day, and ≥4 cups/day of green tea at baseline, respectively, (trend *p* = 0.88).

## 4. Discussion

In this Japanese working population, we observed no cross-sectional or prospective association between green tea consumption and depressive symptoms. To our knowledge, this prospective study is the first to examine associations between green tea consumption and depressive symptoms in a Japanese population.

In previous observational studies, the association between green tea consumption and depressive symptoms has been inconsistent. Of nine studies (eight cross-sectional and one prospective), five studies have observed that green tea consumption was significantly associated with a decrease in depressive symptoms [7,8,12,13] or psychological distress [14], whereas the other three have observed no association with symptoms of depression [9,10] or mental health [15], as did our present study. In the Singapore Longitudinal Ageing Study [11], the only prospective study, the risk of depression (Geriatric Depression Scale ≥5) after a mean of 4 years tended to decrease with green tea consumption, with odds ratios (95% CI) of incident depression of 1.00 (reference), 0.59 (0.21–1.66), and 0.44 (0.60–3.25) for none, <1 cup/day, and ≥1 cup/day, respectively. However, that study included few participants who consumed green tea on daily basis (5%) and did not provide data on the association of greater consumption with depression risk.

Although the reason for the inconsistent findings among studies is not clear, they differed with regard to participant background and assessment of depressive symptoms. For example, study participants were Japanese [9,12,13,14,15], Chinese [7,10], Korean [8], and Singaporean [11], and were elderly [7,9,10,12], middle-aged and older [11,14], middle-aged [13,15], and adults [8]. To assess outcome, they variously used the CES-D [9,13], Geriatric Depression Scale [7,11,12], Patient Health Questionnaire-9 scale [10], self-report of lifetime depression [8], 12-item General Health Questionnaire [15], and Kessler 6-item psychological distress scale [14]. Moreover, the findings were also inconsistent among studies which were conducted in the same country and used the same method to assess outcome. In the present study, frequent drinkers of green tea had greater intake of nutrients that have been suggested to prevent mood disorder (folate, vitamin B6, vitamin B12, n-3 PUFA, magnesium, and zinc). We adjusted for these variables, but the previous studies did not. This may partly explain the inconsistent findings among studies.

As an alternative, this inconsistency may be attributable to differences in the amount of green tea consumption. Niu et al. [12] observed a significantly decrease in odds ratio of having depressive symptoms in participants who drank ≥4 cups/day of green tea compared with those who consumed ≤1 cup/day (0.56, 95% CI 0.39–0.81), but not in those who consumed 2–3 cups/day (0.96, 95% CI 0.66–1.42). Similarly, Hozawa et al. [14] found significantly decreased odds ratio of having psychological distress in the category of ≥5 cups/day of green tea compared with <1 cup/day (0.80, 95% CI 0.70–0.91), but not in the category of 3–4 cups/day (0.89, 95% CI 0.79–1.00) or 1–2 cups/day (0.95, 95% CI 0.86–1.06). Pham et al. [13] reported that the odds ratio for symptoms of depression decreased in the two categories of 2–3 cups/day (0.63, 95% CI 0.39–1.02) and ≥4 cups/day (0.54, 95% CI 0.29–1.00) compared with ≤1 cup/day. Accordingly, a reduction in depressive symptoms seems to be consistent for green tea consumption of ≥4 cups/day. In our study, three-quarters of participants in the highest category (≥2 cups/day) consumed 2–3 cups/day; thus, the odds ratio of developing depressive symptoms for this category largely reflects risk associated with consumption of 2–3 cups of green tea per day. Further investigation of depressive symptoms in relation to greater green tea consumption is required.

Among the strengths of the present study were its prospective design, use of validated questionnaires for depression symptoms and diet, relatively large number of participants, high study participation rate, and adjustment for known or suspected risk factors of symptoms of depression. Our study also had some limitations. The large loss to follow-up could have introduced a degree of selection bias. Nevertheless, we confirmed that the baseline characteristics of participants included and not included in the follow-up were overall similar. Further, because green tea consumption was evaluated using a self-administered questionnaire and at a singly time point only, misclassification due to measurement error is possible. Third, due to small number of participants who drank ≥4 cups/day of green tea, we were unable to examine depression risk associated with high consumption of green tea with sufficient power. Fourth, since we have no data on the preparation, time of the day, and setting of green tea drinking, we cannot control for these variables. Fifth, we used the CES-D scale to assess depressive symptoms and did not examine clinical depression. Accordingly, our findings might not be applicable to clinical depression. Sixth, despite adjusting for major risk factors of depression, we cannot rule out the possibility of bias resulting from unrecognized confounders or residual confounding. Finally, the study participants were all employees of a single company, and the results might not be applicable to general populations.

In summary, we found no cross-sectional or prospective associations between green tea consumption and depressive symptoms. This study provides no evidence supporting the hypothesis that greater consumption of green tea prevents depressive symptoms among Japanese.

## Figures and Tables

**Table 1:** Characteristics of participants by category of green tea consumption.

	Cross-Sectional Analysis (n = 1987)				Longitudinal Analysis (n = 916)			
	<4 Cups/wk	4 Cups/wk to 1 Cup/d	≥2 Cups/d	Trend *p* ^1^	<4 Cups/wk	4 Cups/wk to 1 Cup/d	≥2 Cups/d	Trend *p* ^1^
No. of participants	804	603	580		387	258	271	
Age, year, mean (SD)	40.4 (9.2)	42.5 (10.0)	44.4 (10.8)	<0.001	40.2 (8.8)	42.2 (9.5)	44.1 (9.6)	<0.001
Women, %	8.3	9.5	14.8	<0.001	8.0	9.3	16.2	0.001
Site A, %	51.1	58.7	58.8	0.003	51.4	61.6	62.0	0.005
BMI, kg/m^2^, mean (SD)	23.1 (3.4)	23.3 (3.3)	23.3 (3.3)	0.23	23.0 (3.2)	23.1 (3.3)	22.9 (3.0)	0.90
Married, %	66.5	65.7	65.9	0.78	69.8	72.1	69.7	0.95
Living alone, %	24.4	22.7	18.4	0.010	20.2	17.1	17.3	0.33
Low job grade, %	67.8	69.5	69.5	0.48	68.2	69.0	70.5	0.54
Night and rotating shift work, %	21.4	21.9	12.4	<0.001	20.4	19.0	14.0	0.041
Overtime work ≥30 h/month, %	29.7	26.2	21.9	0.001	25.3	23.6	22.9	0.46
Sleep duration <6 h/day, %	39.3	41.1	40.3	0.66	37.7	36.0	33.2	0.24
Current smoker, %	32.2	27.4	26.2	0.012	32.6	25.2	25.1	0.027
Alcohol consumption ≥1 day/week, %	58.3	52.2	47.1	<0.001	58.1	54.3	46.9	0.005
High job strain ^2^, %	27.5	23.5	23.3	0.062	25.1	24.4	25.1	0.99
High physical activity ^3^,%	26.5	25.9	21.9	0.059	27.1	26.0	21.0	0.084
High leisure-time physical activity ^4^, %	24.3	26.5	25.2	0.64	23.8	24.8	24.7	0.77
History of diabetes, %	2.4	2.2	3.3	0.32	2.8	1.9	1.8	0.38
CES-D score, mean (SD)	12.1 (7.4)	12.3 (7.8)	12.2 (7.7)	0.79	8.5 (4.0)	8.6 (3.9)	8.3 (4.1)	0.55
Total energy intake, kcal/day, mean (SD)	1769 (505)	1827 (583)	1851 (505)	0.004	1736 (500)	1803 (479)	1848 (509)	0.004
Nutrient intake, mean (SD)								
Folate, μg/1000 kcal	142 (51)	159 (51)	199 (66)	<0.001	145 (53)	160 (46)	200 (60)	<0.001
Vitamin B6, mg/1000 kcal	0.59 (0.14)	0.60 (0.14)	0.65 (0.16)	<0.001	0.58 (0.14)	0.60 (0.14)	0.65 (0.14)	<0.001
Vitamin B12, μg/1000 kcal	4.2 (2.1)	4.3 (2.0)	4.8 (2.5)	<0.001	4.1 (2.0)	4.3 (1.9)	4.6 (2.1)	0.002
n-3 PUFA, %energy	1.13 (0.32)	1.17 (0.32)	1.23 (0.36)	<0.001	1.12 (0.30)	1.18 (0.33)	1.21 (0.32)	<0.001
Magnesium, mg/1000 kcal	121 (25)	125 (25)	132 (29)	<0.001	121 (24)	126 (23)	133 (26)	<0.001
Zinc, mg/1000 kcal	4.1 (0.6)	4.2 (0.6)	4.3 (0.6)	<0.001	4.1 (0.6)	4.2 (0.6)	4.2 (0.6)	<0.001
Soft drink consumption ≥4 cups/week, %	22.4	23.2	15.2	0.002	23.5	20.9	16.2	0.025
Coffee consumption ≥1 cup/day, %	66.3	65.2	60.5	0.031	68.7	66.7	60.1	0.026

Abbreviations: CES-D, Center for Epidemiologic Studies Depression; SD, standard deviation. ^1^ Based on the Mantel-Haenszel chi-squared test for categorical variables and linear regression analysis for continuous variables, with ordinal number assigned to category of green tea consumption. ^2^ Job strain ≥0.535 and ≥0.517 (highest quartile) for cross-sectional and longitudinal, respectively. ^3^ Physical activity during housework and work or while commuting ≥18.5 and ≥18.0 METs-hour/day (highest quartile) ≥18.0 for cross-sectional and longitudinal, respectively. ^4^ Leisure-time physical activity ≥10.5 and ≥11.5 METs-hour/week (highest quartile) ≥11.5 for cross-sectional and longitudinal, respectively.

**Table 2:** Odds ratio (95% confidence intervals) of depressive symptoms by category of green tea consumption in cross-sectional and longitudinal associations.

	<4 Cups/Week	4 Cups/Week to1 Cup/Day	≥2 Cups/Day	Trend *p* ^1^
Cross-sectional analysis				
No. of participants	804	603	480	
CES-D ≥16				
No. of cases	221	166	161	
Model 1 ^2^	1.00 (reference)	1.07 (0.84–1.36)	1.13 (0.89–1.45)	0.31
Model 2 ^3^	1.00 (reference)	1.07 (0.83–1.38)	1.19 (0.92–1.55)	0.19
Model 3 ^4^	1.00 (reference)	1.14 (0.88–1.47)	1.29 (0.96–1.73)	0.087
CES-D ≥23 ^5^				
No. of cases	80	64	58	
Model 1 ^2^	1.00 (reference)	1.17 (0.82–1.68)	1.18 (0.81–1.70)	0.36
Model 2 ^3^	1.00 (reference)	1.18 (0.81–1.73)	1.26 (0.85–1.89)	0.24
Model 3 ^4^	1.00 (reference)	1.19 (0.80–1.75)	1.18 (0.75–1.84)	0.43
Longitudinal analysis				
No. of participants	387	258	271	
CES-D ≥16				
No. of cases	62	45	48	
Model 1 ^2^	1.00 (reference)	1.17 (0.77–1.80)	1.27 (0.83–1.95)	0.26
Model 2 ^3^	1.00 (reference)	1.17 (0.73–1.86)	1.26 (0.78–2.03)	0.33
Model 3 ^4^	1.00 (reference)	1.09 (0.68–1.76)	1.12 (0.65–1.91)	0.67
CES-D ≥23 ^5^				
No. of cases	16	14	14	
Model 1 ^2^	1.00 (reference)	1.36 (0.65–2.87)	1.40 (0.66–2.97)	0.37
Model 2 ^3^	1.00 (reference)	1.35 (0.58–3.13)	1.45 (0.61–3.45)	0.38
Model 3 ^4^	1.00 (reference)	1.36 (0.56–3.31)	1.20 (0.45–3.20)	0.69

Abbreviations: CES-D, Center for Epidemiologic Studies Depression. ^1^ Based on multiple logistic regression analysis with assignment of ordinal number to category of green tea consumption. ^2^ Adjusted for age (years), sex, and site. ^3^ Additionally adjusted for body mass index, marital status, living alone, job grade, overtime work, night or rotating shift work, job strain, sleep duration, smoking status, alcohol drinking, leisure-time physical activity, physical activity during work and housework or while commuting to work, under treatment for or history of diabetes, and total energy intake. In longitudinal analysis, CES-D score was additionally adjusted for. ^4^ Additionally adjusted for intake of folate, vitamin B6, vitamin B12, n-3 polyunsaturated fatty acids, magnesium, zinc, soft drink consumption and coffee consumption. ^5^ Participants with a CES-D score of 16-22 were excluded.

## Data Availability

The data presented in this study are available on request from the corresponding author.
